# Association of NOS2A gene polymorphisms with susceptibility to bovine tuberculosis in Chinese Holstein cattle

**DOI:** 10.1371/journal.pone.0253339

**Published:** 2021-06-17

**Authors:** Jun Chai, Qinglu Wang, Bo Qin, Shengkui Wang, Youtao Wang, Muhammad Shahid, Kai Liu, Yifang Zhang, Weijie Qu

**Affiliations:** 1 College of Veterinary Medicine, Yunnan Agricultural University, Panlong District, Kunming, P. R. China; 2 Veterinary Research Institute, Khyber Pakhtunkhwa, Peshawar, Pakistan; Shanghai Jiao Tong University, CHINA

## Abstract

Bovine tuberculosis (bTB) is a global zoonotic disease that has detrimental economic impacts worldwide. The *NOS2A* gene plays a key role in immunological control of many infectious diseases. However, research on the association between NOS2A polymorphisms and bTB infection in Holstein cattle reared on the Yunnan-Guizhou plateau of China is scarce. This study investigated a possible linkage between *NOS2A* polymorphisms and risk of developing bTB in Chinese Holstein cattle. The *NOS2A* gene was genotyped in 144 bTB-infected Holstein cows and 139 healthy controls were genotyped through nucleotide sequencing. Ten single-nucleotide polymorphisms (SNPs) were detected, six of which were associated with susceptibility/resistance patterns of bTB. Furthermore, the C/T genotypes of 671 and 2793, and T/T genotype of E22 (+15) were significantly associated with susceptibility risk; the G/A genotype of 2857, T/T genotype of E9 (+65), and C/C genotype of E9 (+114) probably increased resistance to bTB. In addition, the haplotypes of *NOS2A-2* and *NOS2A-9* were risk factors for bTB susceptibility, while the *NOS2A-5* and *NOS2A-8* haplotypes were contributing protective variants against tuberculosis. There is a significant association between variation in SNPs of *NOS2A* and tuberculosis susceptibility/resistance pattern. These findings suggest that substitution of genetic selection would be helpful for eradicating bTB. However, further investigation is required to study the underlying mechanism through which *NOS2A* polymorphisms affect bTB infection.

## Introduction

Bovine tuberculosis (bTB) is a chronic infectious disease of great concern in cows. The disease is caused by the *Mycobacterium tuberculosis* pathogen. bTB has been documented in many countries, including England, Ireland, Brazil, and China [1–4], where it not only causes large financial losses to the cattle industry but also poses a threat to the health of human beings and wild animals [5]. In developed countries, the prevalence of human tuberculosis (TB) is relatively low, while it remains a potential hazard with an incidence of 10–15% in developing countries due to the high prevalence of bTB [6]. It has been reported that one-third of humans are infected with bTB around the world and 10% of these infected people will develop active TB, suggesting that individual differences may play a critical role in the process of TB infection [7,8].

Nitric oxide (NO) plays a vital role in many physiological and pathological processes, and it is synthesized from the amino acid L-arginine by NO synthase (NOS). NOS is found in three distinct isoforms: endothelial (eNOS), neuronal (nNOS), and inducible (iNOS) [9,10]. iNOS is encoded by the *NOS2A* gene, which is under the transcriptional control of inflammatory mediators produced by immunocompetent cells such as macrophages and neutrophils [11]. iNOS-deficient mice are highly susceptible to TB infection, characterized by atypical granulomas that can facilitate mycobacterial reactivation, dissemination, and transmission [12,13]. Previous studies suggest that the production or activity of iNOS may be influenced by several single nucleotide polymorphisms (SNPs) within the *NOS2A* gene [10,14]. The rs8078340 SNP causes a nucleotide change from guanine to adenine, and this change decreases the amount of bound DNA–protein complex, leading to nitric oxide reduction [15]. Two SNPs (rs1800482, a cytosine to guanine nucleotide change, and rs9282799, a guanine to adenine nucleotide change) in promoter mutations of the *NOS2* gene are associated with a high baseline level of iNOS activity, which increases the synthesis of nitric oxide [16]. The rs57234985 (a thymine to cytosine nucleotide change) SNP is associated with low levels of exhaled nitric oxide [15,17].

Nutritional, environmental and genetic factors are involved in the pathogenesis of TB, and multiple genes control it. Different diseases can be tracked by SNP analysis [18–21]. It has been documented that the rs2779249 and rs2301369 SNPs are associated with TB in Brazilians, and rs2779249, rs9282799, and rs8078340 are associated with the diseased in South African populations [22]. The rs2297518 SNP in exon 16 may act as an important protection factor against development of pulmonary TB in Chinese miners [23]. Therefore, the identification of genetic traits that influence susceptibility or resistance to bTB might offer alternative methods of controlling the disease through genetic selection [24]. However, data regarding the association between *NOS2A* polymorphisms and susceptibility to bTB in Holstein cattle reared on the Yunnan-Guizhou plateau of China are scant. Therefore, we characterized the diversity and SNP variations in the *NOS2A* gene of 144 bTB-infected cattle and 139 healthy controls to help explain the individual differences in susceptibility to bTB.

## Materials and methods

### Ethics

All of the experiments were conducted according to the rules and regulations of the Administration of Affairs Concerning Experimental Animals published by the Ministry of Science and Technology, China, in 2008. All animal experiments were conducted according to the Chinese Guidelines for Institutional Animal Care and Use Committee of the Yunnan Agricultural University (Approval number: IACUC-20132030301).

### Sample collection and TB detection

The dairy cows used for this research were reared on high-altitude (more than 1500 m) farms located in Kunming, Dali, and Yuxi. The samples were collected according to a report of the general investigation of TB in dairy cows by Yunnan Provincial Animal Disease Prevention and Control Center, Dali Animal Disease Prevention and Control Center and Yuxi Animal Disease Prevention and Control Center. A total of 283 Chinese Holstein cows including 144 bTB-positive and 139 bTB-negative (controls) were studied. Blood samples from each experimental cow were collected into a disposable heparin sodium anticoagulation vacutainer.

### Genomic DNA extraction

Genomic DNA was extracted from blood using a Whole Blood Genomic DNA Extraction Kit (Magen Bio, Guangzhou, China) following the manufacturer’s instructions. Samples were subsequently diluted at a concentration of 10 μmol/L and stored at -20°C until use. DNA quality was identified via agarose gel electrophoresis.

### Primer design and synthesis

The primers were designed using Primer Premier 6 software according to the sequence of *NOS2A* (AC_000176.1), published in the National Center for Biotechnology, and synthesized by Takara Company. Information on primers is given in Table 1.

**Table 1:** Primer sequences for the *NOS2A* gene.

Primer	Sequence (5’-3’)	Length	Location	Products (bp)	Annealing temperature (°C)
NOS2A-F1	CTTCTTCCTTGTCCCGTTTCTA	22	Intron1	898	59.6
NOS2A-R1	GAGGGTGGTGTGGAACTAACA	21	Intron2		
NOS2A-F2	GCAGGGGTTTGGTTCAGTTAG	21	Intron5	1004	60.0
NOS2A-R2	CCAGCCTAAAGCCTGCTACATA	22	Intron7		
NOS2A-F3	CTCCAGGTGGGATGAACTTAGA	22	Intron7	932	59.6
NOS2A-R3	TAGTTGCTGCTTAGGGGGAGTA	22	Intron9		
NOS2A-F4	AGCAAAACTGGGCTGTGTTCTA	22	Intron17	904	59.0
NOS2A-R4	AGCCCCTGGTATGTAGGTATGA	22	Intron18		
NOS2A-F5	GGTGAGGTGATGGTGAATGTAG	22	Intron21	908	59.6
NOS2A-R5	GAGGTGAAGGCTCACAGAAGTAA	23	Intron23		

### PCR amplification conditions for *NOS2A*

Samples for PCR were prepared in a volume of 50 μL containing 4.0 μL dNTPs mixture (2.5 mmol/L), 5.0 μL 10×buffer, 0.5 μL Taq polymerase, 1.0 μL each primer (10 mmol/L), 5.0 μL DNA, and 34.5 μL RNase-free water. PCR conditions were as follows: pre-denaturation at 95°C for 5 min, 35 cycles of denaturation at 94°C for 30 s, annealing at 60°C for 30 s, and extension at 72°C for 1 min, with a final elongation at 72°C for 8 min.

### DNA sequencing and detection of polymorphisms

To analyze the PCR amplification products a 1% agarose gel electrophoresis. Products after staining that showed a bright band without nonspecific amplification were sent to Suke Biotechnology (Kunming, China) for sequencing. DNAStar Lasergene 11.1 (Madison, WI, USA) was used to compare the sequences with the whole genome of *NOS2A*.

### Statistical analysis

The χ^2^ test (a = 0.05, *P* > 0.05) was used to determine Hardy-Weinberg equilibrium (HWE) and compare genotype distribution and allele frequency between bTB-infected cattle and controls. Polymorphism information content (PIC) was assessed as follows: PIC > 0.5 (high polymorphism), 0.25 < PIC < 0.5 (moderate polymorphism), and PIC < 0.25 (low polymorphism). The SNPstats software (https://www.snpstats.net) and SPSS v19.0 program (SPSS, Chicago, IL) were used for statistical analysis.

## Results

### Analysis of polymorphisms in the bovine *NOS2A* gene

As shown in Table 2, 10 SNPs were identified in the *NOS2A* gene.

**Table 2:** SNP sites and change in amino acid.

Primer	Site	Variation	Location	Change in amino acid
F1/R1	140	C/G	Exon-2	--
F2/R2	671	C/T	Exon-6	--
F3/R3	E9 (+65)	C/T	Intron-9	--
	E9 (+114)	T/C	Intron-9	--
F4/R4	E18 (-292)	C/T	Intron-17	--
	E18 (-133)	G/A	Intron-17	--
	2243	G/A	Exon-18	--
F5/R5	2793	C/T	Exon-22	--
	2857	G/A	Exon-22	A-T
	E22 (+15)	T/C	Intron-22	--

### Genotype distribution and allele frequency of SNPs in bTB-infected and non-infected cattle

The genotypic and allelic frequencies of all 10 SNPs, except for E9 (+65) in the control group, exceeded up to 5% both in both groups, suggesting that Hardy–Weinberg equilibrium was satisfied (*P >* 0.05). There were two genotypes at loci 140, 671, E18 (-292), E18 (-133), 2243, 2793, and 2857, and three at E9 (+65), E9 (+114), and E22 (+15). PIC values were less than 0.25, indicating low polymorphism in six SNPs (Table 3).

**Table 3:** Distribution of 10 polymorphic genotypes and allele frequency in bTB-infected cattle and controls.

SNPs	Group	Genotype frequency			Allele frequency		PIC	χ^2^	HWE P
		C/C	C/G	G/G	C	G			
140	Infected cattle	135 (0.94)	9 (0.06)	0 (0)	279 (0.97)	9 (0.03)	0.057	0.15	1
	Non-infected cattle	133 (0.96)	6 (0.04)	0 (0)	272 (0.98)	6 (0.02)		0.07	1
		C/C	C/T	T/T	C	T			
671	Infected cattle	126 (0.88)	18 (0.12)	0 (0)	270 (0.94)	18 (0.06)	0.074	0.64	1
	Non-infected cattle	132 (0.95)	7 (0.05)	0 (0)	271 (0.95)	7 (0.03)		0.09	1
		C/C	C/T	T/T	C	T			
E9(+65)	Infected cattle	115 (0.8)	29 (0.2)	0 (0)	259 (0.9)	29 (0.1)	0.177	1.81	0.36
	Non-infected cattle	112 (0.81)	22 (0.16)	5 (0.04)	246 (0.88)	32 (0.12)		6.92	0.019
		C/C	C/T	T/T	C	T			
E9(+114)	Infected cattle	92 (0.64)	47 (0.33)	5 (0.03)	231 (0.8)	57 (0.2)	0.212	0.11	1
	Non-infected cattle	119 (0.86)	20 (0.14)	0 (0)	258 (0.93)	20 (0.07)		0.84	1
		C/C	C/T	T/T	C	T			
E18(-292)	Infected cattle	127 (0.88)	17 (0.12)	0 (0)	271 (0.94)	17 (0.06)	0.106	0.57	1
	Non-infected cattle	124 (0.89)	15 (0.11)	0 (0)	263 (0.95)	15 (0.05)		0.45	1
		G/G	G/A	A/A	G	A			
E18(-133)	Infected cattle	120 (0.83)	24 (0.17)	0 (0)	264 (0.92)	24 (0.08)	0.136	1.19	0.6
	Non-infected cattle	119 (0.86)	20 (0.14)	0 (0)	258 (0.93)	20 (0.07)		0.84	1
		G/G	G/A	A/A	G	A			
2243	Infected cattle	115 (0.8)	29 (0.2)	0 (0)	259 (0.9)	29 (0.1)	0.150	1.81	0.36
	Non-infected cattle	116 (0.83)	23 (0.17)	0 (0)	255 (0.92)	23 (0.08)		1.13	0.6
		C/C	C/T	T/T	C	T			
2793	Infected cattle	118 (0.82)	26 (0.14)	0 (0)	262 (0.91)	26 (0.09)	0.122	1.41	0.6
	Non-infected cattle	126 (0.91)	13 (0.09)	0 (0)	265 (0.95)	13 (0.05)		0.33	1
		G/G	G/A	A/A	G	A			
2857	Infected cattle	144 (1)	0 (0)	0 (0)	288 (1)	0 (0)	0.038		1
	Non-infected cattle	125 (0.9)	14 (0.1)	0 (0)	264 (0.95)	14 (0.05)		0.39	1
		T/T	T/C	C/C	T	C			
E22(+15)	Infected cattle	132 (0.92)	12(0.08)	0 (0.00)	276 (0.96)	12 (0.04)	0.122	0.27	1
	Non-infected cattle	112 (0.81)	25 (0.18)	2 (0.01)	249 (0.9)	29 (0.1)		0.20	0.64

### Association analysis between SNP and bTB susceptibility

Logistic regression was used to investigate the association between single loci of SNP and bTB susceptibility. As shown in Table 4, the SNPs of 671, 2793, E9 (+65), E9 (+114), 2857, and E22 (+15) were significantly associated with susceptibility or resistance to bTB; We also calculated odds ratios and 95% confidence intervals (CIs) (Table 4), and found that the C/T genotype frequencies of both 671 and 2793 were higher in bTB-infected cattle than in controls, indicating that the C/T genotype of these SNPs might be associated with higher susceptibility to bTB. The G/A genotype of 2857 was only found in the control group; therefore, it might confer resistance to bTB.

**Table 4:** Association analysis between SNP sites and tuberculosis infection in cattle.

SNPs	Models	Genotype	bTB-infected cattle	Non-infected cattle	OR (95%CI)	P-value	AIC
140	---	C/C	135 (93.8%)	133 (95.7%)	1.00	0.47	395.7
		C/G	9 (6.2%)	6 (4.3%)	1.48 (0.51–4.27)		
671	---	C/C	126 (87.5%)	132 (95%)	1.00	0.024	391.2
		C/T	18 (12.5%)	7 (5%)	2.69 (1.09–6.67)		
E9(+65)	Recessive	C/C-C/T	144 (100%)	134 (96.4%)	1.00	0.0073	389
		T/T	0 (0%)	5 (3.6%)	NA		
E9(+114)	Log-additive	---	---	---	3.32 (1.90–5.80)	<0.0001	375.9
E18(-292)	---	C/C	127 (88.2%)	124 (89.2%)	1.00	0.79	396.2
		C/T	17 (11.8%)	15 (10.8%)	1.11 (0.53–2.31)		
E18(-133)	---	G/G	120 (83.3%)	119 (85.6%)	1.00	0.6	396
		G/A	24 (16.7%)	20 (14.4%)	1.19 (0.62–2.27)		
2243	---	G/G	115 (79.9%)	116 (83.5%)	1.00	0.43	395.6
		G/A	29 (20.1%)	23 (16.6%)	1.27 (0.69–2.33)		
2793	---	C/C	118 (81.9%)	126 (90.7%)	1.00	0.032	391.6
		C/T	26 (18.1%)	13 (9.3%)	2.14 (1.05–4.35)		
2857	---	G/G	144 (100%)	125 (89.9%)	1.00	<0.0001	375.6
		G/A	0 (0%)	14 (10.1%)	NA		
E22(+15)	Log-additive	---	---	---	0.38 (0.19–0.76)	0.0039	387.9

To explore potential genotypic risk factors for TB, we analyzed genetic models using Akaike’s information criterion We found that locus E9 (+65) showed recessive inheritance and the T/T genotype of E9 (+65) was only found in the control group, suggesting that these genotypes are probably associated with resistance to bTB. The best genetic model of locus E9 (+114) was log-additive inheritance, and the C/C genotype frequency of E9 (+114) in bTB-infected cattle (64.0%) was lower than control (86.0%), suggesting that these genotypes were protective against TB. The best genetic model for locus E22 (+15) was log-additive inheritance, and the T/T genotype frequency of E22 (+15) in bTB-infected cattle (92.0%) was higher than that of controls (81.0%) as shown in Table 4.

### Haplotype analysis

We identified and analyzed haplotypes based on *NOS2A* gene polymorphism and discarded total occurrence frequencies below 0.01. *NOS2A-2*, *NOS2A-5*, *NOS2A-8*, and *NOS2A-9* had significant associations with bTB susceptibility/resistance (*P* < 0.05). Further analysis revealed that the frequency of haplotype *NOS2A-2* (OR, 3.97; 95% CI, 1.85–8.55) was significantly higher in bTB-infected cattle (*P* = 0.1098) than in controls (*P* = 0.0479), and that of *NOS2A-9* was only found in bTB-infected cattle (*P* = 0.1098), suggesting that these two haplotypes are risk factors for bTB. In contrast, the haplotype frequency of *NOS2A-5* (OR, 0.24; 95% CI, 0.24) was significantly higher in controls (*P* = 0.0577) and that of *NOS2A-8* was only found in controls, indicating that these two haplotypes are protective factors against TB (Table 5).

**Table 5:** Association analysis between *NOS2A* haplotypes and bTB infection.

NO.	Haplotypes	Freq of bTB-infected cattle	Freq of non-infected cattle	Freq	OR (95%CI)	P-value
NOS2A-1	CCCCCGGCGT	0.4977	0.5644	0.4836	1	--
NOS2A-2	CCCTCGGCGT	0.1098	0.0479	0.0923	3.97 (1.85–8.55)	0.0005
NOS2A-3	CCTCCGGCGT	0.0558	0.0532	0.0629	1.05 (0.48–2.29)	0.9
NOS2A-4	CCCCCGACGT	0.0488	0.0315	0.0506	2.48 (0.88–6.97)	0.086
NOS2A-5	CCCCCGGCGC	0.0059	0.0577	0.0461	0.24 (0.08–0.72)	0.012
NOS2A-6	CCCCCGGTGT	0.043	0.0235	0.0429	2.51 (0.89–7.04)	0.083
NOS2A-7	CCCCCAGCGT	0.0192	0.0264	0.0306	0.77 (0.23–2.61)	0.68
NOS2A-8	CCCCCGGCAT	NA	0.0282	0.0239	0.08 (0.01–0.71)	0.024
NOS2A-9	CTCCCGGCGT	0.0246	NA	0.023	0.00 (-Inf—Inf)	1
NOS2A-10	CCCCTGGCGT	0.0096	0.0144	0.0174	0.70 (0.12–3.96)	0.69

## Discussion

Polymorphism in the *NOS2A* gene is associated with many diseases, including cerebral palsy, malaria, type 2 diabetes mellitus, asthma, rheumatoid arthritis, purpura, and coronary heart disease [25–32]. In addition, recent findings have demonstrated that the SNP locus polymorphism of the *NOS2A* gene as well as the polymorphism of the microsatellite promoter is associated with TB. Velez *et al*. found that the *NOS2A* and *TLR2* genes regulate epistatic interactions as modifiers of SLC11A1-mediated TB risk in African Americans and Caucasians [33]. Moller *et al*. reported that haplotypes composed of two SNP loci in the *NOS2A* gene promoter, rs9282799 and rs8078340, are significantly associated with TB in South Africans [22]. In addition, the *NOS2A* gene promoter region (CCTTT) has a significant protective effect against TB in various Indian populations [34]. A study African Americans found that multiple SNPs in the *NOS2A* gene were associated with TB, and some sites were also found to have synergistic effects on the *TLR4* and *IFNGR1* genes and affect TB risk [15].

The *NOS2A* gene regulates the NO levels by encoding inducible NOS (iNOS) and mediating immune responses to TB and other infectious diseases. iNOS is a pivotal regulator of susceptibility to tuberculosis infection in mice [35]. It has been documented that NO has the direct cytotoxicity to mycobacteria [36,37]. Therefore, the *NOS2A* gene is associated with bTB susceptibility.

In the past decade, many studies have investigated the relationship between the *NOS2A* gene and human TB, but there is insufficient research on the correlation between *NOS2A* polymorphisms and bTB susceptibility, especially on the plateau of China. Therefore, we studied this topic in Holstein cattle reared on the Yunnan Plateau. We found that SNP 2857 caused an amino acid change from alanine to threonine, and that PIC values of 10 SNPs were less than 0.25, suggesting low polymorphism.

Furthermore, haplotypes *NOS2A-2* and *NOS2A-9* were found to be risk factors for bTB susceptibility, while the *NOS2A-5* and *NOS2A-8* were considered to protect against bTB. The occurrence and development of bTB is affected by various factors including pathogens, environmental causes, and susceptibility genes. Our results offer information that could improve breeding for bTB-resistant cattle, potentially eradicating the disease over time. Meanwhile, *NOS2A* polymorphisms could be potential biomarkers for predicting bTB risk. A multi-model deep learning framework based on convolutional neural networks, which has been verified for the prediction of risk in Alzheimer’s disease and gastric cancer [38,39], may be used to aid in the development of the prediction model. Moreover, the exact mechanisms involved bTB susceptibility and resistance need to be verified in further experiments. For example, during TB infection, the gut microbiota is modulated and may be responding to immunological changes in the host, as the *NOS2A* gene is involved in the immunological regulation of many infectious diseases. Therefore, the relationship between NOS2A polymorphisms and the gut microbiome during bTB infection could be investigated further using a metagenomic analysis tool [40]. Meanwhile, mRNA modifications are potentially new insights into this biological basis of bTB susceptibility. The recent progress in N4-Acetylcytidine on RNA expression is also playing key role on a lot of diseases, and people have found that the *NOS2A* abnormal expression is mediated through mRNA N4-Acetylcytidine [41].

This study also has its limitation. Firstly, the *NOS2A* polymorphisms associated with bTB in this study were intend to be analyzed using meta-analysis, which has been reported in several publications [42–45], but the sample size limitation in this study prevented the application of meta-analysis. Secondly, causal effects of this gene’s variants on the risk of bTB should be explored through integrating gene expression data under the Mendelian Randomization framework [46,47], to determine if the genetic variants may affect the bTB susceptibity through regulating the gene’s expression level. Unfortunately, the expression data is not available.

## Conclusion

The C/T genotypes of 671 and 2793 and T/T genotype of E22 (+15) may be associated with susceptibility to bTB; the G/A genotype of 2857, T/T genotype of E9 (+65), and C/C genotype of E9 (+114) probably increase resistance to bTB. In addition, the haplotypes of *NOS2A-2* and *NOS2A-9* may be risk factors for bTB, and those of *NOS2A-5* and *NOS2A-8* may be protective factors against bTB. However, the underlying causality and mechanisms are unclear; therefore, further investigation and confirmation are required.

## Abbreviations

AIC: Akaike’s information criterion
bTB: Bovine Tuberculosis
eNOS: endothelial nitric oxide synthase
iNOS: inducible nitric oxide synthase
nNOS: neuronal nitric oxide synthase
NO: Nitric oxide
NOS: NO synthase
PIC: Polymorphism information content
SNP: Single nucleotide polymorphism
TB: Tuberculosis
TST: Tuberculin skin test

## References

[1] Bermingham ML, More SJ, Good M, Cromie AR, Higgins IM, Brotherstone S, et al. Genetics of tuberculosis in Irish Holstein-Friesian dairy herds. J Dairy Sci. 2009;92(7):3447–56. doi: 10.3168/jds.2008-1848 . 19528623

[2] Evans JT, Smith EG, Banerjee A, Smith RM, Dale J, Innes JA, et al. Cluster of human tuberculosis caused by Mycobacterium bovis: evidence for person-to-person transmission in the UK. Lancet. 2007;369(9569):1270–6. doi: 10.1016/S0140-6736(07)60598-4 . 17434402

[3] Liu W, Cao WC, Zhang CY, Tian L, Wu XM, Habbema JDF, et al. VDR and NRAMP1 gene polymorphisms in susceptibility to pulmonary tuberculosis among the Chinese Han population: a case-control study. Int J Tuberc Lung Dis. 2004;8(4):428–34. . 15141734

[4] Leandro ACCS, Rocha MA, Lamoglia-Souza A, VandeBerg JL, Rolla VC, Bonecini-Almeida MdG. No association of IFNG+874T/A SNP and NOS2A-954G/C SNP variants with nitric oxide radical serum levels or susceptibility to tuberculosis in a Brazilian population subset. BioMed research international. 2013;2013:901740. doi: 10.1155/2013/901740 . 24024215 PMC3759278

[5] Pullen MF, Boulware DR, Sreevatsan S, Bazira J. Tuberculosis at the animal-human interface in the Ugandan cattle corridor using a third-generation sequencing platform: a cross-sectional analysis study. BMJ Open. 2019;9(4):e024221. doi: 10.1136/bmjopen-2018-024221 . 30962227 PMC6500349

[6] Kemal J, Sibhat B, Abraham A, Terefe Y, Tulu KT, Welay K, et al. Bovine tuberculosis in eastern Ethiopia: prevalence, risk factors and its public health importance. BMC Infect Dis. 2019;19(1):39. doi: 10.1186/s12879-018-3628-1 . 30630431 PMC6327393

[7] Aravindan PP. Host genetics and tuberculosis: Theory of genetic polymorphism and tuberculosis. Lung India. 2019;36(3):244–52. doi: 10.4103/lungindia.lungindia_146_15 . 31031349 PMC6503728

[8] Yu ZG, Wang BZ, Li J, Ding ZL, Wang K. Association between interleukin-17 genetic polymorphisms and tuberculosis susceptibility: an updated meta-analysis. Int J Tuberc Lung Dis. 2017;21(12):1307–13. doi: 10.5588/ijtld.17.0345 . 29297452

[9] Han D-H, Kim M-S, Shin H-S, Park KP, Kim H-D. Association between periodontitis and salivary nitric oxide metabolites among community elderly Koreans. J Periodontol. 2013;84(6):776–84. doi: 10.1902/jop.2012.120237 . 22799757

[10] Wang Z, Feng K, Yue M, Lu X, Zheng Q, Zhang H, et al. A non-synonymous SNP in the NOS2 associated with septic shock in patients with sepsis in Chinese populations. Hum Genet. 2013;132(3):337–46. doi: 10.1007/s00439-012-1253-4 . 23192595

[11] Kendall HK, Marshall RI, Bartold PM. Nitric oxide and tissue destruction. Oral Dis. 2001;7(1). . 11354916

[12] MacMicking JD, North RJ, LaCourse R, Mudgett JS, Shah SK, Nathan CF. Identification of nitric oxide synthase as a protective locus against tuberculosis. Proceedings of the National Academy of Sciences of the United States of America. 1997;94(10):5243–8. doi: 10.1073/pnas.94.10.5243 . 9144222 PMC24663

[13] Reece ST, Loddenkemper C, Askew DJ, Zedler U, Schommer-Leitner S, Stein M, et al. Serine protease activity contributes to control of Mycobacterium tuberculosis in hypoxic lung granulomas in mice. The Journal of clinical investigation. 2010;120(9):3365–76. doi: 10.1172/JCI42796 . 20679732 PMC2929725

[14] de O S Mansur T, Gonçalves FM, Martins-Oliveira A, Speciali JG, Dach F, Lacchini R, et al. Inducible nitric oxide synthase haplotype associated with migraine and aura. Mol Cell Biochem. 2012;364(1–2):303–8. doi: 10.1007/s11010-012-1231-0 . 22234503

[15] Velez DR, Hulme WF, Myers JL, Weinberg JB, Levesque MC, Stryjewski ME, et al. NOS2A, TLR4, and IFNGR1 interactions influence pulmonary tuberculosis susceptibility in African-Americans. Hum Genet. 2009;126(5):643–53. doi: 10.1007/s00439-009-0713-y . 19575238 PMC2881538

[16] Kun JF, Mordmüller B, Perkins DJ, May J, Mercereau-Puijalon O, Alpers M, et al. Nitric oxide synthase 2(Lambaréné) (G-954C), increased nitric oxide production, and protection against malaria. J Infect Dis. 2001;184(3):330–6. doi: 10.1086/322037 . 11443559

[17] Levesque MC, Hobbs MR, O’Loughlin CW, Chancellor JA, Chen Y, Tkachuk AN, et al. Malaria severity and human nitric oxide synthase type 2 (NOS2) promoter haplotypes. Hum Genet. 2010;127(2):163–82. doi: 10.1007/s00439-009-0753-3 . 19859740 PMC2939908

[18] Kamath PL, Foster JT, Drees KP, Luikart G, Quance C, Anderson NJ, et al. Genomics reveals historic and contemporary transmission dynamics of a bacterial disease among wildlife and livestock. Nature communications. 2016;7:11448. doi: 10.1038/ncomms11448 . 27165544 PMC4865865

[19] Wang X, Jiao X, Tian Y, Zhang J, Zhang Y, Li J, et al. Associations between maternal vitamin D status during three trimesters and cord blood 25(OH)D concentrations in newborns: a prospective Shanghai birth cohort study. Eur J Nutr. 2021. doi: 10.1007/s00394-021-02528-w . 33661376

[20] Chen J, Zhao X, Cui L, He G, Wang X, Wang F, et al. Genetic regulatory subnetworks and key regulating genes in rat hippocampus perturbed by prenatal malnutrition: implications for major brain disorders. Aging (Albany NY). 2020;12(9):8434–8458. doi: 10.18632/aging.103150 . 32392183 PMC7244046

[21] Yan X, Zhao X, Li J, He L, Xu M. Effects of early-life malnutrition on neurodevelopment and neuropsychiatric disorders and the potential mechanisms.Prog Neuropsychopharmacol Biol Psychiatry. 2018;83:64–75. doi: 10.1016/j.pnpbp.2017.12.016 . 29287829

[22] Möller M, Nebel A, Valentonyte R, van Helden PD, Schreiber S, Hoal EG. Investigation of chromosome 17 candidate genes in susceptibility to TB in a South African population. Tuberculosis (Edinb). 2009;89(2):189–94. doi: 10.1016/j.tube.2008.10.001 . 19147409

[23] Qu Y, Tang Y, Cao D, Wu F, Liu J, Lu G, et al. Genetic polymorphisms in alveolar macrophage response-related genes, and risk of silicosis and pulmonary tuberculosis in Chinese iron miners. Int J Hyg Environ Health. 2007;210(6):679–89. doi: 10.1016/j.ijheh.2006.11.010 . 17223386

[24] le Roex N, van Helden PD, Koets AP, Hoal EG. Bovine TB in livestock and wildlife: what’s in the genes? Physiol Genomics. 2013;45(15):631–7. doi: 10.1152/physiolgenomics.00061.2013 . 23757394

[25] Garme Y, Moudi M, Saravani R, Galavi H. Nitric Oxide Synthase 2 Polymorphisms (rs2779248T/C and rs1137933C/T) and the Risk of Type 2 Diabetes in Zahedan, Southeastern Iran. Iran J Public Health. 2018;47(11):1734–41. . 30581791 PMC6294872

[26] Porojan MD, Cătană A, Popp RA, Dumitrascu DL, Bala C. The role of NOS2A -954G/C and vascular endothelial growth factor +936C/T polymorphisms in type 2 diabetes mellitus and diabetic nonproliferative retinopathy risk management. Ther Clin Risk Manag. 2015;11:1743–8. doi: 10.2147/TCRM.S93172 . 26664124 PMC4669928

[27] Li H, Wang X, Lu X, Zhu H, Li S, Duan S, et al. Co-expression network analysis identified hub genes critical to triglyceride and free fatty acid metabolism as key regulators of age-related vascular dysfunction in mice. Aging (Albany NY). 2019;11(18):7620–7638. doi: 10.18632/aging.102275 . 31514170 PMC6781998

[28] Somuncu MN. Inspection of Endothelial Nitric Oxide Synthase Gene Polymorphism in Patients With Henoch Schnlein Purpura. Arch Rheumatol. 2014;29(2):73–80.

[29] Negi VS, Mariaselvam CM, Misra DP, Muralidharan N, Fortier C, Charron D, et al. Polymorphisms in the promoter region of iNOS predispose to rheumatoid arthritis in south Indian Tamils. Int J Immunogenet. 2017;44(3):114–21. doi: 10.1111/iji.12315 . 28374504

[30] Hirai K, Shirai T, Suzuki M, Shimomura T, Itoh K. Association between (CCTTT)n repeat polymorphism in NOS2 promoter and asthma exacerbations. J Allergy Clin Immunol. 2018;142(2). doi: 10.1016/j.jaci.2018.02.023 . 29518423

[31] Dzodzomenyo M, Ghansah A, Ensaw N, Dovie B, Bimi L, Quansah R, et al. Inducible nitric oxide synthase 2 promoter polymorphism and malaria disease severity in children in Southern Ghana. PloS one. 2018;13(8):e0202218. doi: 10.1371/journal.pone.0202218 . 30118498 PMC6097674

[32] Torres-Merino S, Moreno-Sandoval HN, Thompson-Bonilla MDR, Leon JAO, Gomez-Conde E, Leon-Chavez BA, et al. Association Between rs3833912/rs16944 SNPs and Risk for Cerebral Palsy in Mexican Children. Mol Neurobiol. 2019;56(3):1800–11. doi: 10.1007/s12035-018-1178-6 . 29931509 PMC6394613

[33] Velez DR, Hulme WF, Myers JL, Stryjewski ME, Abbate E, Estevan R, et al. Association of SLC11A1 with tuberculosis and interactions with NOS2A and TLR2 in African-Americans and Caucasians. Int J Tuberc Lung Dis. 2009;13(9):1068–76. . 19723394 PMC2902362

[34] Jena M, Srivastava AK, Singh RK, Sharma PR, Das PK, Bamezai RNK. NOS2A promoter (CCTTT)n association with TB lacks independent functional correlation amongst Indians. Tuberculosis (Edinb). 2014;94(1):81–6. doi: 10.1016/j.tube.2013.10.004 . 24291065

[35] Cooper AM, Pearl JE, Brooks JV, Ehlers S, Orme IM. Expression of the nitric oxide synthase 2 gene is not essential for early control of Mycobacterium tuberculosis in the murine lung. Infect Immun. 2000;68(12):6879–82. doi: 10.1128/IAI.68.12.6879-6882.2000 . 11083808 PMC97793

[36] Chan J, Xing Y, Magliozzo RS, Bloom BR. Killing of virulent Mycobacterium tuberculosis by reactive nitrogen intermediates produced by activated murine macrophages. J Exp Med. 1992;175(4):1111–22. doi: 10.1084/jem.175.4.1111 . 1552282 PMC2119182

[37] Long R, Light B, Talbot JA. Mycobacteriocidal action of exogenous nitric oxide. Antimicrob Agents Chemother. 1999;43(2):403–5. doi: 10.1128/AAC.43.2.403 . 9925545 PMC89090

[38] Yu H, Pan R, Qi Y, Zheng Z, Li J, Li H, et al. LEPR hypomethylation is significantly associated with gastric cancer in males. Exp Mol Pathol. 2020;116:104493. Epub 2020/07/14. doi: 10.1016/j.yexmp.2020.104493 . 32659237

[39] Liu M, Li F, Yan H, Wang K, Ma Y, Alzheimer’s Disease Neuroimaging I, et al. A multi-model deep convolutional neural network for automatic hippocampus segmentation and classification in Alzheimer’s disease. Neuroimage. 2020;208:116459. Epub 2019/12/15. doi: 10.1016/j.neuroimage.2019.116459 . 31837471

[40] Zheng S, Zhao T, Yuan S, Yang L, Ding J, Cui L, et al. Immunodeficiency Promotes Adaptive Alterations of Host Gut Microbiome: An Observational Metagenomic Study in Mice. Front Microbiol. 2019;10:2415. Epub 2019/11/30. doi: 10.3389/fmicb.2019.02415 . 31781050 PMC6853035

[41] Jin G, Xu M, Zou M, Duan S. The Processing, Gene Regulation, Biological Functions, and Clinical Relevance of N4-Acetylcytidine on RNA: A Systematic Review. Mol Ther Nucleic Acids. 2020; 20:13–24. doi: 10.1016/j.omtn.2020.01.037 . 32171170 PMC7068197

[42] Wu Y, Cao H, Baranova A, Huang H, Li S, Cai L, et al. Multi-trait analysis for genome-wide association study of five psychiatric disorders. Transl Psychiatry. 2020;10(1):209. Epub 2020/07/02. doi: 10.1038/s41398-020-00902-6 . 32606422 PMC7326916

[43] Jiang L, Wang K, Lo K, Zhong Y, Yang A, Fang X, et al. Sex-Specific Association of Circulating Ferritin Level and Risk of Type 2 Diabetes: A Dose-Response Meta-Analysis of Prospective Studies. J Clin Endocrinol Metab. 2019;104(10):4539–51. Epub 2019/05/11. doi: 10.1210/jc.2019-00495 . 31074789

[44] Xu MQ, Ye Z, Hu FB, He L. Quantitative assessment of the effect of angiotensinogen gene polymorphisms on the risk of coronary heart disease. Circulation. 2007;116(12):1356–66. Epub 2007/09/12. doi: 10.1161/CIRCULATIONAHA.107.728857 . 17846284

[45] Xu M, Lin Z. Genetic influences of dopamine transport gene on alcohol dependence: a pooled analysis of 13 studies with 2483 cases and 1753 controls. Prog Neuropsychopharmacol Biol Psychiatry. 2011;35(5):1255–60. doi: 10.1016/j.pnpbp.2010.11.001 . 21078357 PMC5335908

[46] Zhang F, Baranova A, Zhou C, Cao H, Chen J, Zhang X, et al. Causal influences of neuroticism on mental health and cardiovascular disease. Human Genetics. 2021. doi: 10.1007/s00439-021-02288-x . 33973063

[47] Zhang F, Rao S, Cao H, Zhang X, Wang Q, Xu Y, et al. Genetic evidence suggests posttraumatic stress disorder as a subtype of major depressive disorder. J Clin Invest. 2021. doi: 10.1172/JCI145942 . 33905376 PMC8803333

